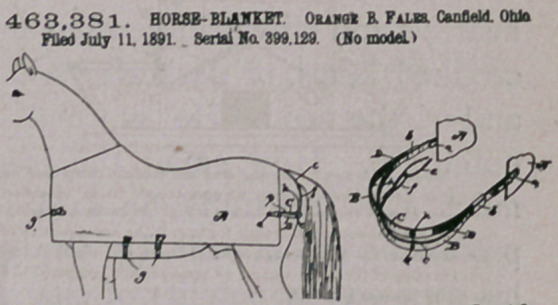# Recent Patents

**Published:** 1892-02

**Authors:** 


					﻿RECENT PATENTS
RELATING TO
VETERINARY MEDICINE AND ANIMAL INDUSTRY.
Issued by U. S. Patent Office ending January, 1892.
Claim —The herein-described shoe, the same berng divided from
its toe to its quarters to form a lower spring-tread, the spring-
tread being tbiokeued at its front to form toe-calk and upon its
upper side at its from a flange 4*, said tread being narrowed at its
opposite sides to expose the nail-hoies^jn the shoe, substantially as
specified.
Claim.—1. A crib for storing feed and feeding cattle and hogs,
having an elevated inclined floor, an open-topped .lateral extension at
the lower edge of the inclined floor, a trough for cattle in the top por-
tion of said extension, and a trough for hogs io the lower portion of the
said extension, the latter trough arranged to receive the feed dropped
over the edge.of the upper trough, all arranged and combined to oper
ate in the manner set forth.
2. A crib having an elevated floor inclined in opposite directions
from its center to produce the roof of a pig-sty, open-topped latera
extensions at the sides of the crib and sty, troughs for feeding cattle,"
extended through the top portions of the said extensions and the wall,
of the crib, having openings leading thereto to allow feed to descend
from th^ inclines of the floor into said troughs, and troughs for feed
ing hogs, extended through the lower portions of the said extensions
and.adapted to receive the waste dropped over the edges of the upper
troughs by the cattle, all arranged and combined in the manner set
forthL for the purposes stated
CZarm.—in a horseshoe-nail clincher, the combination of the hau-
dle A, provided at one end with a stationary jaw c and with parallel
lugs or projections B B, the short movable jaw 0, pivotally supported
between said lugs, the spring D, arranged between the jaws C and e*
to normally separate the same, and the lever E, pivoted between the
lugs B B on the outer side of the movable jaw C and provided with
a projecting pointed end f to impinge on said movable jaw and force
it toward the stationary jaw when the bandies or levers are drawn
apart* substantially as described
Claim.—I. A horsehoe formed with a channel in its under side, a.
cushion or filling material in said channel, said shoe having a series of
openings around its upper side communicating with the channel, aud
the clips extending from the edges of said openings, substantially as
described.
2. The horseshoe described, consisting of a plate of sheet meta)
bent to form a channel with sides b’ b\ said shoe having a top piece
with a series of openings therein and an angular end and with clips
extending from the edges of the opening, the said sides b' b1 being bent
about the angular ends of the top piece, substantially .as described.
Claim.—1. The combination of the blank and the loop, said blank
having perforations narrower than said loop, and said loop having It*
ends beveled, inserted part way through said perforations, and clinohM
upon the under side of said blank, substantially as described.
2. The combination of the perforated blank and the loop having
the beveled and pointed ends, said loop being wider .than said perfo--
rations, and said loop ends being wedged in said perforations and
clinched upon the under side of said blank, substantially as described^
Claim.—I. Ao improved horse-clean*r conitrtiog of a handle, a
rod extending in alignment therewith and having an enlarged head,
a aria of wires applied to said head and arranged aroood said rod
parallel thereto and attached to a sliding disk, and an adjustable
straioing device connected with the handle and disk, whereby ths
wires are drawn and held taut, substantially a* shoen and described
2. la a horse cleaner, the combination of the handle A, the
threaded forked rod B, provided with the nut C and perforated plate
D, the disk E, haring a threaded central aperture a. the rod F, pro-
vided with the hemispherical head G, and the wires H. attached to
the disk D and extooding over the hemispherical head G. substan-
tially as described.
Claim.—Io a feed-trough, the combination at the end boards aod
inclined pieces b b D D g p, central partition C, swinging receptacle B.
and racks E E, hinged as set forth, the whole arranged as sbowo and
deeeribed.
prrm — | A saddle-tree having a strenthening-bndge a upon the
under side side, thereof, the continuous raised portions c e the sbool-
dere i i, and the bp j, as and for the purpose specified.
2	A saddle-tree having a strengthening-bridge a upon Che unde*
side thereof, the continuous raised portions e e, and the flanges d aod
f thereon, as aod for the purpose set forth
3	A saddle-tree having a strengthening-bridge a upon its under
■de. the continuous raised portions c r. the flanges d and /. the bridge*
A 6 on its lower extremities, and the flanges 4 on the bridges b b. a*
sod for the purpose specified
Claim.— 1 The combination with the horseshoe having an aper-
ture, of the expansible calk having the shank located in said aperture
aod the expanding pin or wedge extending longitudinally within the
calk from the contact eod thereof and being disconnected and out or
engagement with the shoe, so as to be forced inwardly by the concus-
eioo on its lower eod, substantially as set forth and described, whereby
the shank is maintained expanded within said aperture of the shoe.
2. The combination, with a horseshoe having an aperture, of the
calk having a shank to fit to said aperture aod provided with the smooth
exterior, said calk being longitudinally divided into similar sections
with the flat meeting faces and a longitudinal passagb extending from
the contact end of the calk throughout the length thereof, and the
expanding-pin having the straight sides longitudinally located within
said passage out of engagement with the shoe aod extending from the
contact eod of the calk, so that the pin will be constantly forced in-
wardly by the concussion.
C2atm.—). The combination of the blanket A, the straps B aod 0,
the springs D, fixed to the straps 0 and D, aod the ptercing-etnds c,
fixed to the strap BjSnbstantislly as aod for th* purpose specified.
				

## Figures and Tables

**Figure f1:**
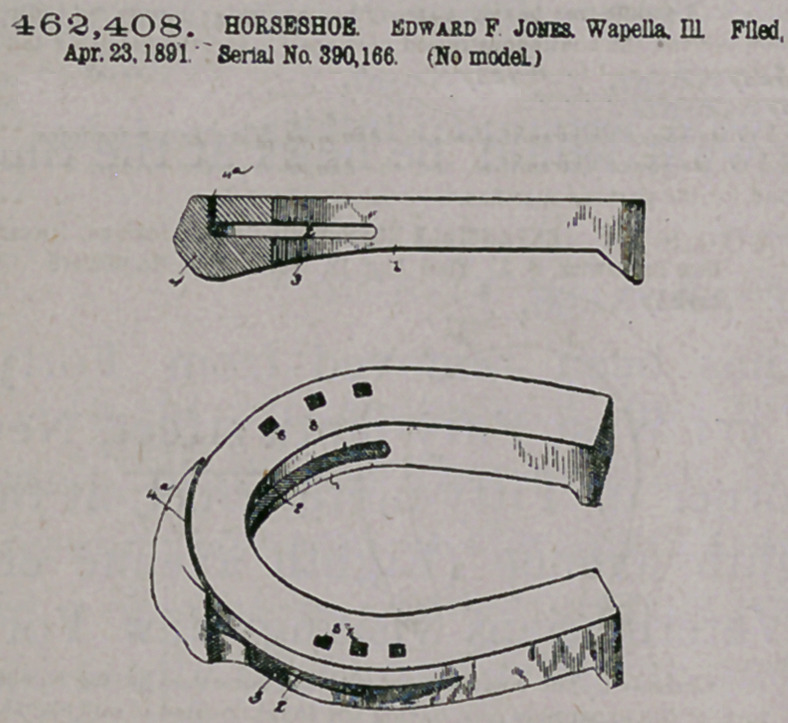


**Figure f2:**
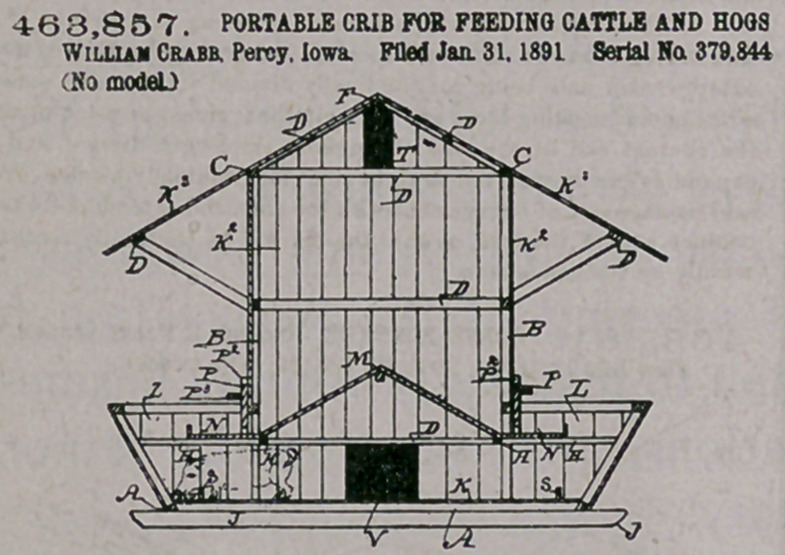


**Figure f3:**
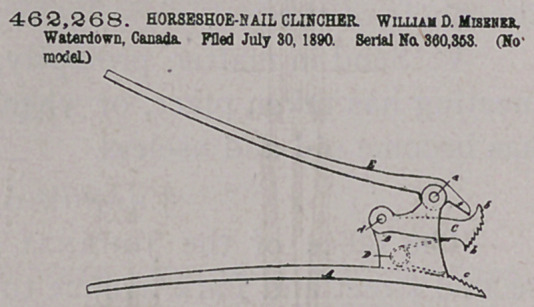


**Figure f4:**
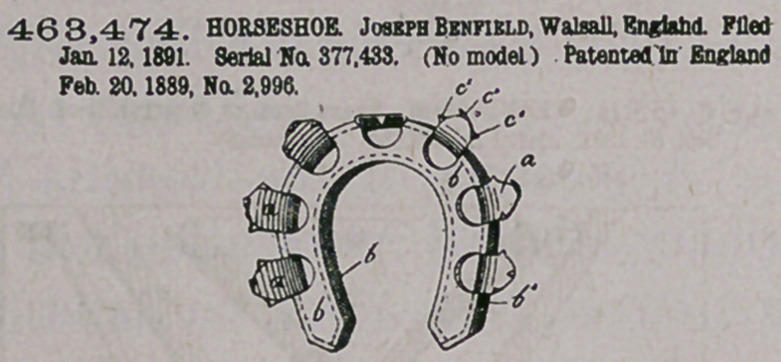


**Figure f5:**
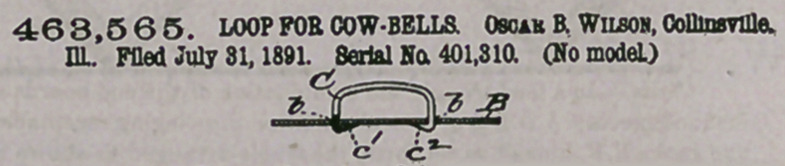


**Figure f6:**
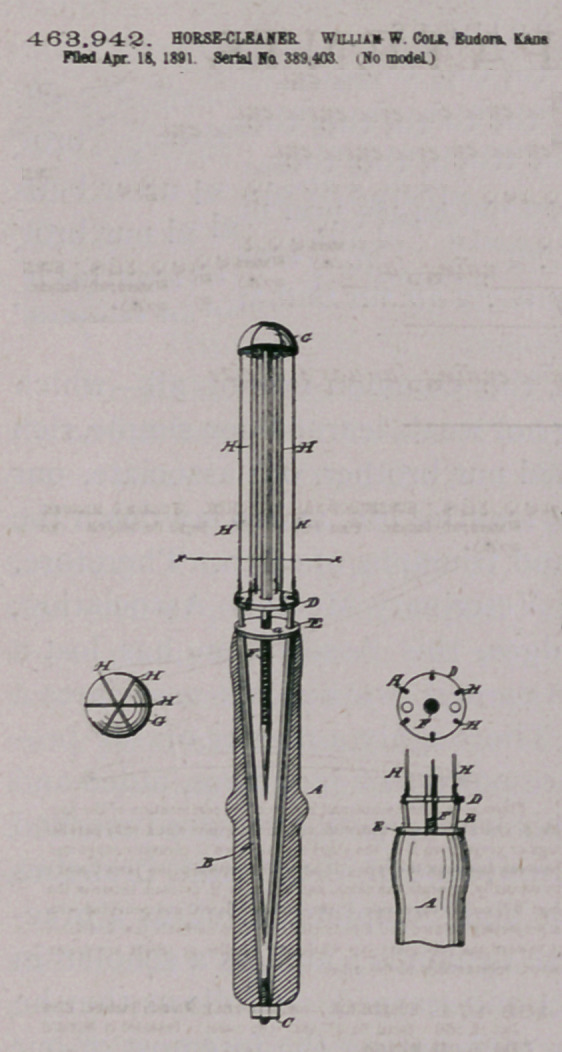


**Figure f7:**
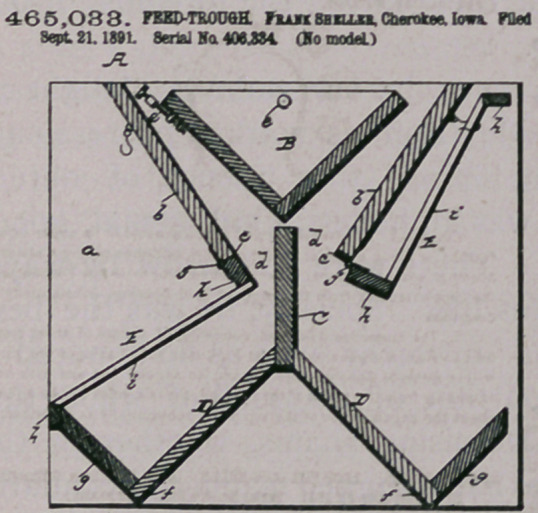


**Figure f8:**
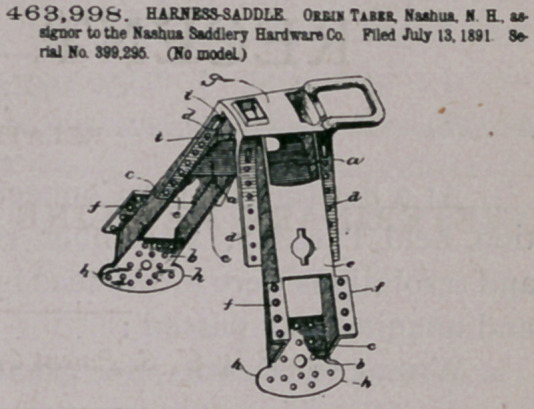


**Figure f9:**
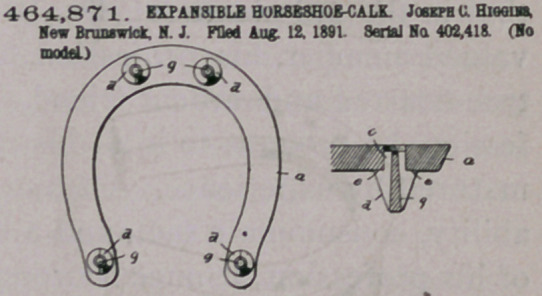


**Figure f10:**